# Differential Responses of Thalamic Reticular Neurons to Nociception in Freely Behaving Mice

**DOI:** 10.3389/fnbeh.2016.00223

**Published:** 2016-11-21

**Authors:** Yeowool Huh, Jeiwon Cho

**Affiliations:** ^1^Center for Neural Science, Korea Institute of Science and TechnologySeoul, South Korea; ^2^Department of Neuroscience, University of Science and TechnologyDaejeon, South Korea

**Keywords:** thalamic reticular nucleus, thalamocortical circuit, nociception, extracellular single unit recording, mice, awake recording, formalin test

## Abstract

Pain serves an important protective role. However, it can also have debilitating adverse effects if dysfunctional, such as in pathological pain conditions. As part of the thalamocortical circuit, the thalamic reticular nucleus (TRN) has been implicated to have important roles in controlling nociceptive signal transmission. However studies on how TRN neurons, especially how TRN neuronal subtypes categorized by temporal bursting firing patterns—typical bursting, atypical bursting and non-bursting TRN neurons—contribute to nociceptive signal modulation is not known. To reveal the relationship between TRN neuronal subtypes and modulation of nociception, we simultaneously recorded behavioral responses and TRN neuronal activity to formalin induced nociception in freely moving mice. We found that typical bursting TRN neurons had the most robust response to nociception; changes in tonic firing rate of typical TRN neurons exactly matched changes in behavioral nociceptive responses, and burst firing rate of these neurons increased significantly when behavioral nociceptive responses were reduced. This implies that typical TRN neurons could critically modulate ascending nociceptive signals. The role of other TRN neuronal subtypes was less clear; atypical bursting TRN neurons decreased tonic firing rate after the second peak of behavioral nociception and the firing rate of non-bursting TRN neurons mostly remained at baseline level. Overall, our results suggest that different TRN neuronal subtypes contribute differentially to processing formalin induced sustained nociception in freely moving mice.

## Introduction

Pain serves a critical role for survival by alerting for danger. However, when the normal pain system becomes dysfunctional, as is in chronic pathological pain, pain serves an adverse effect and can cause serious debilitation. In thalamic pain syndrome, a type of chronic pain, patients with lesion in the thalamus develop pathological pain symptoms (Gonzales et al., 1992; Parrent et al., 1992; Jeanmonod et al., 1993; Barraquer-Bordas et al., 1999; Kim et al., 2007; Klit et al., 2009). The extent of thalamic lesion in these patients often includes the thalamic reticular nucleus (TRN; Gonzales et al., 1992; Jeanmonod et al., 1993), implying that the TRN may have a key role in regulating pain signal transmission.

The TRN is a structure that is solely composed of GABAergic inhibitory neurons (Jones, 2007). As part of the thalamus, the TRN receives input from the cortex and other thalamic nuclei and provides major inhibitory input to each thalamic nucleus (Jones, 2007). Since the thalamus is a structure where most sensory information, including nociception, is transmitted, sensory modulation is thought to occur at the thalamic level before reaching the cortex (McCormick and Feeser, 1990; Sherman, 2001; Ab Aziz and Ahmad, 2006). Specifically the ability of a single relay thalamic neuron to switch firing between single spikes or burst of high frequency spikes, called tonic and burst firing, respectively, is suggested to be important for modulating sensory information (McCormick and Feeser, 1990; Sherman, 2001; Lee et al., 2005). The switch from tonic firing to burst firing occurs by inhibition of thalamic neurons via the presence of T-type Ca^2+^ channels, which is primed for activation only after the membrane has been hyperpolarized (Jahnsen and Llinás, 1984a,b; Destexhe et al., 1998). Activation of T-type Ca^2+^ channels triggers thalamocortical neurons to fire in low threshold spike (LTS) bursts (Jahnsen and Llinás, 1984a,b; Destexhe et al., 1998). By providing major inhibitory input to the sensory thalamus, the TRN could influence sensory thalamic neurons to fire in burst modes (Le Masson et al., 2002; Halassa et al., 2011), and therefore, will be important for modulating sensory signals relayed in the thalamus.

The TRN has been implicated in various functions such as regulating brain rhythms (Steriade and Deschenes, 1984; Steriade et al., 1985; Steriade and Llinás, 1988; von Krosigk et al., 1993; Fuentealba and Steriade, 2005; Halassa et al., 2011), attention (Guillery et al., 1998; Wimmer et al., 2015), and sensory modulation (Lee et al., 1994a,b). Nociceptive signals may also be modulated by TRN. However, few studies have investigated the relationship between TRN neuronal activity and nociceptive signal processing (Peschanski et al., 1980; Montagne-Clavel and Olivéras, 1995; Yen and Shaw, 2003), especially in terms of TRN neuronal firing modes in the awake state. Studying the response of TRN neurons to nociception in the awake state may be important for understanding nociceptive signal processing since TRN neuronal activity is greatly influenced by different arousal states, with burst firing becoming more prevalent during sleep or anesthesia, while tonic firing is dominant in the awake state (Barrionuevo et al., 1981; Domich et al., 1986; Steriade et al., 1986).

Physiologically, TRN neurons can be divided into bursting and non-bursting neurons via the presence or absence of T-type Ca^2+^ channels, respectively (Brunton and Charpak, 1997; Fuentealba and Steriade, 2005). Bursting neurons can be sub-divided into typical and atypical bursting neurons, based on the temporal firing pattern and waveform shape (Lee et al., 2007). The typical burst firing pattern of TRN neurons is characterized by a greater number of burst spikes than that of thalamocortical neurons and by an acceleration then a deceleration pattern of inter-spike interval (ISI) of burst spikes (Domich et al., 1986; Steriade et al., 1986). Atypical burst firing pattern, on the other hand, is characterized by fewer burst spikes than typical bursts and gradually increasing ISI of burst spikes (Lee et al., 2007). Typical bursting activity of TRN neurons was demonstrated to be important for generating sleep cycles (Domich et al., 1986; Steriade et al., 1993), brain rhythms (Steriade and Deschenes, 1984; Steriade et al., 1985; Steriade and Llinás, 1988; von Krosigk et al., 1993; Fuentealba and Steriade, 2005), and regulating attention (Guillery et al., 1998; Wimmer et al., 2015), while the roles of atypical bursting and non-bursting TRN neurons were less obvious (Lee et al., 2007). Likewise, different neuronal types of TRN are likely to contribute differentially to nociceptive signal processing, but no study has yet investigated their functional significance in nociceptive signal processing, especially in behaving animals.

In this study, we investigated how different TRN neuronal types respond differentially to formalin induced sustained nociception in freely moving mice. TRN is reported to have a loosely topographical organization, thus, we targeted the TRN region corresponding to the hind paw pad, where formalin was injected, for extracellular single unit recording in behaving mice.

## Materials and Methods

### Animals

Male mice (First generation C57BL/6J × 129/SvJae hybrid, 10–15 weeks old, body weight 26–32 g) were used for the experiment. Mice were housed at constant temperature (22 ± 1°C) with free access to food and water under a 12:12 h light and dark cycle (light cycle beginning at 8:00 AM). Mice were group caged (2–5 mice per cage) before microdrive implant surgery and single caged after the surgery. All experiments were in accordance and guidance of the Korea Institute of Science and Technology Animal Care and Use Committee (Approval number: AP 201326). All surgical procedures were done under general anesthesia (Zoletil) and sufficient level of anesthesia was maintained throughout the surgery. The condition of animals was monitored every day after surgery. To minimize stress, animals were handled gently before and during experiments.

### Microdrive Implantation Surgery

Microdrives (Neuralynx Inc., Bozeman, MT, USA) with four tetrodes were surgically implanted into the anterior dorsal TRN (AP: −0.60 mm, ML: −1.38 mm, DV: −3.10 mm; (Paxinos and Franklin, 2001) for extracellular single unit recordings in freely moving mice. Each tetrode was four wires (12.5 μm nichrome wire with polyamide-insulation, Kanthal Precision Technology, Minneapolis, MN, USA) intertwined into one electrode. The electrode tip was gold plated to obtain an impedance around 400–500 kΩ. The anterior dorsal TRN was chosen for recording because it is the region reported to have a somatotopic correspondence to the hind paw in rats (Shosaku et al., 1984; Yen and Shaw, 2003) and nociception was induced in the hind paw in our study.

For microdrive implantation surgery, mice were anesthetized with Zoletil (30 mg/kg body weight, intra peritoneal injection) and a supplementary dose, one third of the initial dose, was given to maintain a sufficient level of anesthesia. Anesthetized mice were fixed onto a stereotaxic instrument (David Kopf Instruments, Tujunga, CA, USA) and craniotomy was performed with a drill above the target region. Four stainless screws were screwed into the skull, two in the frontal skull, one in the parietal skull, and one in the occipital skull, to provide support to anchor the microdrive. Once tetrodes were positioned in the target TRN, microdrives were fixed by filling in dental cement between the skull and the microdrive. Mice were allowed to recover for at least a week and condition of mice were monitored every day during recovery.

### Formalin Induced Nociceptive Behavior and Extracellular Single Unit Recording

Formalin was used to induce tonic nociception. Behavioral responses and neuronal activity changes to nociception were simultaneously measured. Mice were habituated to the experimental setting in the recording chamber with recording cables attached for 30 min each day for at least a week, including the test day. Experimental room was set to be 22 ± 1°C in temperature with a white noise generator on at maximum 85 dB. Recording chamber was a white opaque plastic cylinder (diameter: 20 cm; height: 25 cm) placed on top of a beveled mirror for unobstructed behavioral monitoring.

Nociception was induced by injecting 10 μl of formalin (5%, 1:20 dilution of 37% formalin solution in deionized water) subcutaneously to the left hind paw pad with a syringe (Hamilton, Mercer, NJ, USA). Formalin dose was chosen based on a previous study reporting that 5% formalin induced the greatest nociception related behaviors (Okuda et al., 2001). Behavioral and neuronal activities were recorded simultaneously for 10 min before formalin injection and for 1 h right after formalin injection. Neuronal signals were acquired extracellularly with an analog Cheetah Acquisition System (Neuralynx Inc., Bozeman, MT, USA). Signals were amplified, filtered and sampled at 30,303 Hz. Level of behavioral nociception was quantified by summing the duration of licking and shaking behavior of the left paw in 5 min segments. Measurements of two investigators were averaged.

### Histology

Recording sites were verified with histology. After the completion of experiments mice were overdosed with 2% avertin and passed 20–50 μA DC current for 10 s to make electrolytic lesions at the recording location. Mice were then transcardially perfused with physiological saline (0.9%) followed by 10% formalin solution diluted in saline (1:10 dilution of 37% formaldehyde solution in 0.9% saline). Brains were extracted and stored in 10% formalin solution diluted in deionized water (1:10 dilution of 37% formaldehyde solution) for a day. Afterwards, brains were transferred to a 30% sucrose solution and stored at 4°C for a week before sectioning. Coronal sections (50 μm) were made with a microtome (Microm, Germany). Sections were stained with cresyl violet (Sigma, Billerica, MA, USA) and examined under a light microscope to identify recording locations.

### Neuronal Signal Analysis and TRN Neuron Subtypes

Neuronal signals acquired with Cheetah Acquisition System were spike sorted into single-units using the SpikeSort3D program provided from Neuralynx Inc., Bozeman, MT, USA. Well isolated signals from the SpikeSort3D program were further confirmed to be from single units with cross-correlation and ISI histograms. Among the well isolated signals, only the ones confirmed to be recorded in TRN with histology were analyzed.

**Figure 1:**
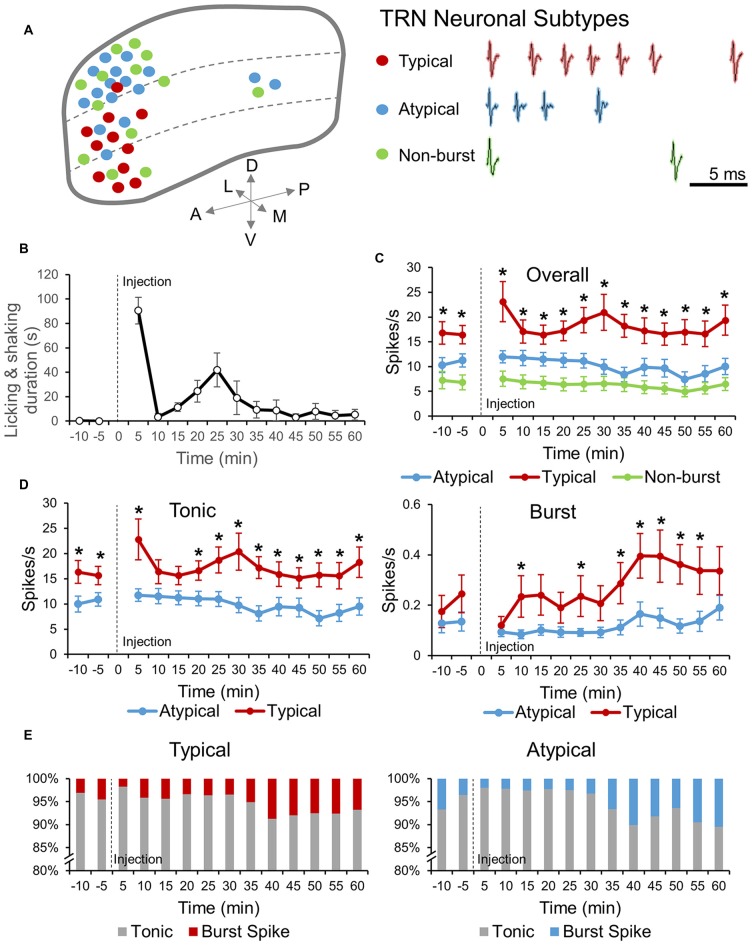
**Thalamic reticular nucleus (TRN) neuronal subtypes and response to formalin induced nociception. (A)** Distribution of the three TRN neuronal subtypes recorded within the TRN (left). Red, blue and green dots represent, typical, atypical and non-burst TRN neurons, respectively. Sample of burst firing patterns of typical and atypical TRN neurons and spiking pattern of a non-burst TRN neuron are shown in right. **(B)** Behavioral nociceptive responses before and after subcutaneous injection of formalin (5%, 10 μl) in the left paw pad (*n* = 7 mice). **(C)** Neuronal activities of before and after formalin injection for three different TRN neuronal sub-types: Atypical (*n* = 14 neurons, 5 mice), Typical (*n* = 10 neurons, 5 mice) and Non-burst (*n* = 14 neurons, 6 mice). One-way analysis of variance (ANOVA) with Games Howell *post hoc* was used to compare firing rate differences between neuronal subtypes at each time segment. Significance was determined at *P < 0.05. **(D)** Tonic and burst spike firing rate changes before and after formalin for atypical and typical TRN neurons (same neurons as in **C**). Two-tailed *t*-test was used to compare means at each time segment. Significance was determined at *P < 0.05. **(C,D)** All data points are Mean ± SEM. Repeated measures ANOVA was used to test for within group firing rate changes over time after formalin injection and all groups had significant changes over time. **(E)** Relative changes in the ratio of tonic and burst spikes over time for atypical (left) and typical (right) TRN neuronal type. Abbreviations: A, anterior; P, posterior; D, dorsal; V, ventral; L, lateral; M, medial.

Upon completion of the recordings, signals from single neurons were categorized into typical, atypical and non-bursting TRN neurons based on the presence of each burst firing pattern or absence of any bursts after visual inspection of individual spike trains with NeuroExplorer 4 (Nex Technologies, Littleton, MA, USA). Visual inspection revealed that an individual neuron had a tendency to generate only one type of burst firing pattern. Samples of a typical and atypical burst firing pattern are delineated in Figure 1A. Distinction between typical and atypical burst firing neuron is based on the presence or absence of an acceleration then deceleration of burst spike firing, respectively. Since accelerating or decelerating pattern of bursts cannot be determined from two spikes firing in high frequency, any neurons that only had two spikes firing in high frequency were classified as non-bursting neuron. In addition, firing rate of high frequency two-spike occurring in the neurons classified as non-bursting showed no formalin induced changes over time, suggesting that there were no burst modulation in these neurons. Based on previous electrophysiological studies of TRN neurons (Domich et al., 1986; Contreras et al., 1992; Kimura et al., 2012) and our own observations, burst spikes were defined to be spikes firing in high frequency within ≤8 ms in the first ISI and then within ≤10 ms ISI of subsequent spikes after ≥100 ms proceeding silent period. All the other spikes not defined to be burst spikes were considered to be tonic spikes.

Changes in firing rate before and after formalin injection of overall, tonic and burst firings were analyzed in 5 min segments. To compare changes in firing rate relative to the baseline, firing rates of each neuronal type were normalized by the following method: (firing rate after formalin injection −baseline firing rate)/(firing rate after formalin injection + baseline firing rate). The magnitude of the changes in firing rate relative to the baseline is not reflected in this normalization method, but this method provides an accurate representation of relative neuronal activity changes over time induced by formalin: positive values indicate an increase, while negative values indicate a decrease in firing rate relative to the baseline.

Burst firing property changes induced by formalin was also analyzed for typical and atypical bursting neurons. Changes in the average number of burst spikes composing a burst (burst spikes/burst), length of bursts, interval between bursts (inter-burst-interval: IBI), interval between burst spikes (intra-burst-interval: IntraBI) and a period of silence before and after a burst, were analyzed over time in 5 min segments.

One-way analysis of variance (ANOVA) was used to compare means between TRN neuronal subtypes. Repeated measures ANOVA with Games Howell *post hoc* was used to test for significance of within group firing rate changes over time. To compare differences between the baseline and formalin injection after normalization, one sample *t*-test was used. Two tailed *t*-test was used to compare means between typical and atypical neurons. Significance was determined at *P* < 0.05 for all statistical tests.

## Results

### TRN Neuronal Subtypes and Responses to Formalin Induced Nociception

To investigate the relationship between TRN neuronal subtypes and nociceptive signal modulation, TRN neuronal activity changes before and after formalin induced nociception were recorded in freely moving mice. Recordings mainly targeted the anterior dorsal TRN, the location which has somatotopic correspondence to the hind paw (Shosaku et al., 1984), where nociception was induced in the present study.

Locations of TRN neuronal subtypes recorded in our experiments were delineated in Figure 1A (left). Typical, atypical and non-bursting TRN neuronal subtypes were distinguished based on the pattern of bursts generated or absence of any burst (see “Materials and Methods” Section for detail). Representative burst spiking patterns of typical and atypical TRN neurons and spike shape of non-bursting TRN neuron were shown in Figure 1A (right). Burst spike frequencies of typical neurons accelerated then decelerated while those of atypical neurons only decelerated. Typical neurons also had a characteristic spike shape distinct from other neuronal types (Supplementary Figure 1). Distribution of neuronal subtypes within the TRN was non-homogenous. Typical neurons were mainly located ventrally while atypical neurons were mainly located dorsally. Non-bursting neurons also had a tendency to be located in the dorsal side, but also found in the ventral side.

TRN neuronal activities and behavioral responses to formalin induced nociception were recorded simultaneously. Recordings were paused at the point of formalin injection and restarted immediately after the injection. Thus, acute nociception due to a needle penetrating the skin was not included in this study. Behaviorally, mice showed characteristic biphasic nociceptive response to formalin with peaks at 0–5 min and 20–25 min, and an interphase of low nociceptive responses separating the two peaks at 5–10 min (Figure 1B). Neuronal activity before and after formalin injection were different among TRN neuronal subtypes (Figure 1C). The overall firing rate of typical TRN neurons was significantly higher than those of the other neuronal subtypes (*P* = 0.026), while the overall firing rate of atypical neurons was not significantly different from that of non-bursting neurons (*P* = 0.137). The overall firing rate of all three TRN neuronal subtypes changed significantly over time when analyzed with repeated measures ANOVA, suggesting that all three subtypes had temporal modulations in response to nociception.

Since the ability of TRN neurons to switch between tonic and burst firing modes was suggested to have key roles in sensory modulation (Hartings et al., 2003), tonic and burst firing rate changes induced by formalin were analyzed for typical and atypical TRN neurons (Figure 1D). Tonic firing rates of typical and atypical neurons were significantly different before and after formalin injection. Specifically, typical neurons had significantly greater tonic firing rate than that of atypical neurons (Figure 1D, left). The most distinct difference between typical and atypical TRN neurons was that the tonic firing of typical neurons changed biphasically, similar to the pattern of behavioral nociception changes (Figure 1D, left). Atypical neurons, on the other hand, hardly changed their tonic firing rate until after the second peak of behavioral nociception, when tonic firing rate gradually decreased (Figure 1D, left). Furthermore, burst firing rate changes of typical and atypical neurons were also distinct (Figure 1D, right). The baseline burst firing rate of the two neuronal types did not differ and formalin injection had a tendency to inhibit burst firing of both neuronal types. However, after the initial decrease, burst firing rate of typical neurons rapidly started to increase and continued to increase above the baseline, which was similar to the burst firing pattern of sensory thalamic neurons in response to formalin induced nociception (Huh et al., 2012). In addition, the burst firing rate of typical neurons, after formalin injection, was significantly greater than that of atypical neurons at most time segments. The burst firing rate of atypical neurons, conversely, remained low after formalin injection until it increased towards the end of the recording.

Changes in the relative percentage of tonic and burst spikes for typical and atypical TRN neurons were analyzed over time to investigate whether they differed between the two neuronal subtypes (Figure 1E). The majority of the spikes were tonic spikes for both typical and atypical neurons and the pattern of burst spikes proportion changes over time of typical and atypical neurons were similar. During the baseline recording, the proportion of burst spikes were 4.5% for typical neurons and 3.6% for atypical neurons right before formalin injection. After formalin injection, the proportion of burst spikes decreased to 1.8% for typical neurons and 2.0% for atypical neurons. Overtime, the proportion of burst spikes increased, with the maximum occurring at 30–40 min for typical neurons (8.7%) and at 55–60 min for atypical neurons (10.5%).

### TRN Neuronal Subtypes Respond Differentially to Formalin Induced Nociception

**Figure 2:**
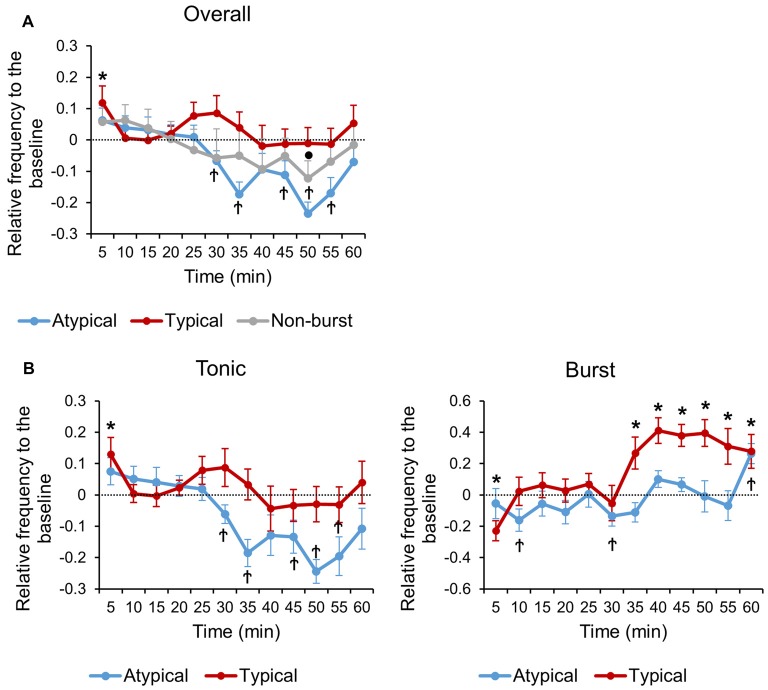
**Normalized neuronal activity changes relative to the baseline of each TRN neuronal subtypes. (A)** Relative changes in overall firing rate over time of each TRN neuronal subtypes. **(B)** Relative changes in tonic and burst firing rate over time of typical and atypical TRN neurons. **(A,B)** All data points are Mean ± SEM. Horizontal line at zero indicates the baseline level for each neuronal subtype. One sample tailed *t*-test was used to compare means of each TRN neuronal type with respective baseline at each time segment. Significance was determined at *P < 0.05 for typical, ^†^*P* < 0.05 for atypical and ^•^*P* < 0.05 for non-burst TRN neuron.

Firing rates of TRN neuronal subtypes were normalized to emphasize the temporal changes of individual neurons relative to respective baselines (see “Materials and Methods” Section for detail). The three TRN neuronal subtypes responded very distinctively to formalin induced nociception over time (Figure 2A), while the arousal state remained constant (Supplementary Figure 2). The overall firing rate of typical neurons never dropped below the baseline. Interestingly, it increased and decreased in a biphasic pattern, precisely matching the biphasic pattern of behavioral nociception. Typical neurons significantly increased overall firing rate at 0–5 min and showed a tendency to increase at 20–25 min (*P* = 0.053), which corresponds exactly to the two peaks of the behavioral nociception. The overall firing rate of atypical neurons, in contrast, showed a tendency to increase at 0–5 min (*P* = 0.120), maintained baseline level until it started to significantly decrease at 25–30 min, corresponding to right after the second peak of behavioral nociception, then remained significantly lower than baseline at most time segments. In contrast, the overall firing rate of non-bursting neurons was relatively constant, remaining around baseline level throughout the recording period except at the 45–50 min time segment, when it decreased significantly below the baseline.

Tonic and burst firing rate changes, relative to the baseline, of typical and atypical TRN neurons were also investigated to better reveal temporal change patterns (Figure 2B). Tonic firing of typical neurons increased biphasically with peaks at 0–5 min (*P* = 0.027) and 20–25 min (*P* = 0.064; Figure 2B, left). Tonic firing of atypical neurons showed a tendency to increase at the 0–5 min segment (*P* = 0.091), maintained baseline level, and then decreased significantly below the baseline after the 25–30 min segment (Figure 2B, left).

The burst firing rate of typical neurons decreased significantly at the 0–5 min segment, maintained at baseline level through 5–30 min, and then remained significantly increased above the baseline level through 30–60 min (Figure 2B, right). The time segments where burst firing of typical neurons significantly increased above the baseline level corresponds to the time segments after the second peak of behavioral nociception when the level of behavioral nociception started to decrease and stayed low (Figure 1B). Burst firing of atypical neurons, in contrast, remained relatively constant at baseline level until it significantly increased above the baseline level at 55–60 min (Figure 2B, right). Normalized results showed a more specific temporal correlation between neuronal firing rates and behavior.

### Burst Firing Property Differences Between Typical and Atypical TRN Neurons

**Figure 3:**
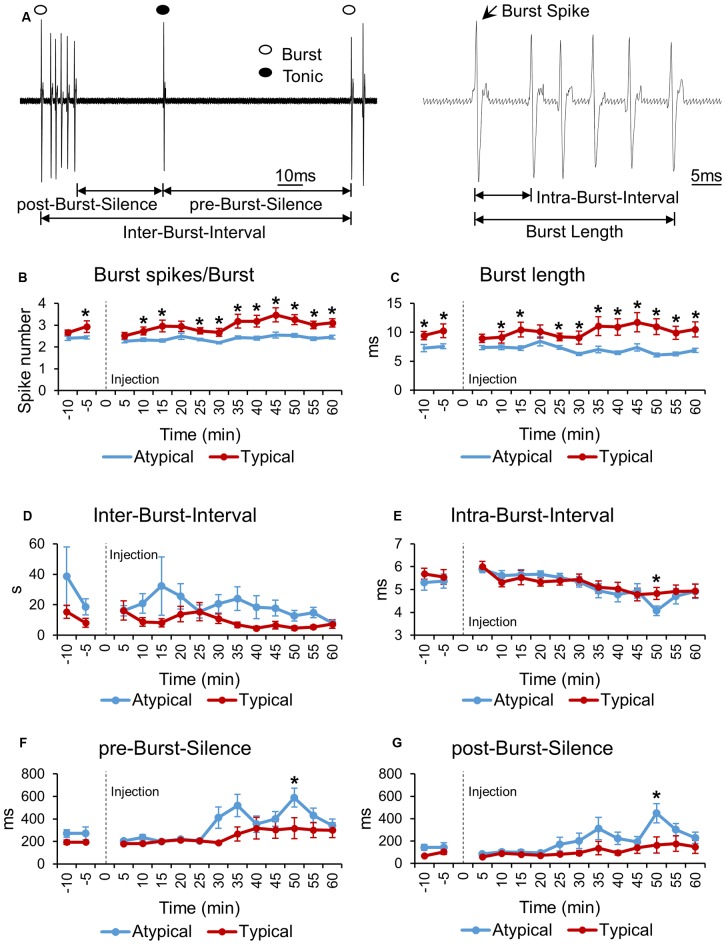
**Bursting property changes induced by formalin of typical and atypical TRN neurons. (A)** Schematic drawing illustrating the components used for burst property analysis. **(B–G)** Bursting property changes of typical and atypical TRN neurons before and after formalin injection analyzed in 5 min segments. All data points are Mean ± SEM. Two-tailed *t*-test was used to compare means between the two TRN neuronal subtypes at each time segment. Significance was determined at *P < 0.05. Repeated measures ANOVA was used to test for within group firing rate changes over time after formalin injection.

Burst firing properties of sensory thalamic neurons have been reported to change accordingly to changes in behavior of formalin induced nociception (Huh et al., 2012). Since the burst firing activity of sensory thalamic neurons could be initiated by burst firing of TRN neurons (Steriade et al., 1993; Pinault et al., 2001), burst firing properties of TRN may also be important. In addition, different types of bursts generated by different TRN neuronal subtypes may contribute differentially to nociceptive signal modulation. Therefore, we investigated changes in burst firing properties, before and after formalin induced nociception, over time for typical and atypical TRN neurons. Burst firing properties investigated in the present study are depicted in Figure 3A.

First of all, the number of burst spikes composing a burst was investigated for typical and atypical TRN neurons (Figure 3B). Typical TRN neurons had a greater average number of burst spikes composing a burst than atypical TRN neurons had at almost all time segments. For typical neurons, the minimum number of burst spikes occurred at 0–5 min, 2.52 burst spikes per burst, while for atypical neurons the minimum occurred at 25–30 min, 2.19 burst spikes per burst. The maximum number of burst spikes occurred at 40–45 min for both neuronal subtypes: 3.47 burst spikes per burst for typical neurons and 2.54 burst spikes per burst for atypical neurons. The number of burst spikes per burst of typical neurons changed significantly over time while that of atypical neurons did not change (repeated measures ANOVA, typical: *F* = 7.486, *P* = 0.04; atypical: *F* = 1.93, *P* = 0.12). Likewise, the burst length of typical neurons was significantly longer than that of atypical neurons (Figure 3C).

However, IBI between typical and atypical TRN neurons was not different (Figure 3D). In addition, the IntraBI, defined as the terval between burst spikes, of typical and atypical neurons was also similar and relatively consistent over time, except at one time segment, 45–50 min, when IntraBI of atypical neurons significantly decreased (Figure 3E). This time segment corresponds to when the tonic firing of atypical neurons was the lowest (Figure 2B).

As a rough measure of pre-burst inhibition and after burst hyperpolarization, length of silences before and after a burst, pre-burst-silence and post-burst-silence, respectively, were analyzed over time (Figures 3F,G). The length of pre- and post-burst-silences of typical TRN neurons were similar over time. The length of pre- and post-burst-silence of atypical neurons, however, had a tendency to increase after 25–30 min, which corresponds to the second peak of the behavioral nociceptive responses, and became significantly longer than those of typical neurons at 45–50 min. Increase in pre- and post-burst-silences of atypical neurons are likely due to significant decrease in the overall firing of atypical neurons after 25–30 min (Figure 2A).

## Discussion

Our study showed that different TRN neuronal subtypes responded differentially to formalin induced nociception in freely moving mice. Typical neurons had the most robust response to nociception and non-bursting neurons showed the least modulation to nociception. Tonic firing pattern of typical TRN neurons mirrored the changes in behavioral nociception, while that of atypical TRN neurons remained at baseline level until after the second peak of behavioral nociception, at which point it decreased significantly below baseline. Burst firing pattern of typical and atypical neurons also changed distinctively. For example, burst firing of typical neurons was inhibited right after formalin injection, but soon recovered to baseline and eventually became significantly potentiated above baseline after the second peak of behavioral nociception, which corresponds to low behavioral nociception level. In contrast, burst firing of atypical neurons remained at baseline until towards the end of the recording, at which point it increased significantly above baseline. These results imply that different TRN neuronal subtypes contribute differentially to nociceptive signal processing.

In the context of the role of TRN within the thalamocortical circuit, i.e., receiving inputs from the sensory thalamus and the cortex and providing inhibition to the sensory thalamus, typical TRN neurons are likely to have the greatest influence in modulating activity of sensory thalamic neurons. Our previous studies that investigated the relationship between sensory thalamic neuronal firing modes and nociceptive signal processing, showed that burst firing of sensory thalamic neurons was associated with anti-nociception (Huh et al., 2012; Huh and Cho, 2013). Since burst firing of sensory thalamic neurons may occur only after inhibition, via the presence of T-type Ca^2+^ channels which is primed for activation only after membrane hyperpolarization (Jahnsen and Llinás, 1984a,b; Destexhe et al., 1998), inhibitory TRN input is crucial for generating bursts in the sensory thalamus. Consequently, TRN could be the key component in modulating ascending nociceptive signals.

Of the TRN neuronal subtypes, typical neurons may be the subtype that has the greatest influence in modulating nociceptive signals, as the activity of typical neurons exhibited the most dramatic changes that were temporally correspondent to behavioral nociception, during formalin induced nociception. In addition, typical bursts generated by these neurons are more likely to generate bursting in sensory thalamic neurons because typical bursts have significantly greater number of burst spikes per burst than atypical bursts. Bursts with greater number of burst spikes are suggested to ensure reliable signal transmission by having greater temporal integration power (Lisman, 1997; Swadlow and Gusev, 2001) and many studies have demonstrated that typical burst firing of TRN neurons generates burst firing in sensory thalamic neurons (Steriade et al., 1993; Pinault et al., 2001).

Notably, changes in burst firing of typical TRN neurons were tightly correlated with the changes in behavioral nociceptive responses. For example, burst firing of typical neurons increased significantly above the baseline right after the second peak of behavioral nociception, which corresponds to when behavioral nociceptive responses are reduced. The time when burst firing of typical TRN neurons significantly increase precedes the time when burst firing of sensory thalamic neurons increase (Huh et al., 2012), indicating that burst firing of sensory thalamic neurons is initiated by burst firing of typical TRN neurons. This strongly suggests that activity of typical TRN neurons may play a key role in attenuating sustained nociception by initiating the generation of bursts in the sensory thalamus to obstruct nociceptive signal transmission.

Tonic firing of typical TRN neurons, on the other hand, increased biphasically in a manner that mirrored the biphasic changes of the behavioral nociceptive responses. The role of tonic firing typical TRN neurons may be to provide sustained inhibition to maintain activity of sensory thalamic neurons at a certain level to protect the neurons from being over-activated. A study showed that tonic firing of neurons in perigeniculate nucleus (PGN), structure analogous to the visual TRN in higher order animals, activated GABA_A_ receptors while burst firing of PGN neurons activated GABA_B_ receptors in the sensory thalamus (Kim et al., 1997). Likewise, tonic firing of typical TRN neurons may also preferentially activate GABA_A_ receptors of sensory thalamic neurons. Since inhibition by activation of GABA_A_ receptors is shorter lasting than GABA_B_ receptors (Bormann, 1988; Kim et al., 1997), activation of GABA_A_ receptors will be advantageous in maintaining neurons to fire at a certain level. In this regard, tonic firing of typical TRN neurons may act as a buffer to control sensory thalamic neurons from being over-activated.

Activity of atypical TRN neurons, in contrast, showed completely different changes compared to that of typical TRN neurons. For example, tonic firing of atypical TRN neurons remained relatively constant after formalin injection until it significantly decreased below the baseline after the second peak of behavioral nociception. Therefore, inhibition provided by atypical neurons will have weakened after that time. Burst firing of atypical neurons, however, significantly increased towards the end of the recording period, implying that atypical neurons may also contribute to the generation of bursts in the sensory thalamus.

The role of non-bursting TRN neurons in modulating nociceptive signals was less obvious, as the activity of these neurons remained relatively constant after formalin injection. Thus, the functional significance of these neurons in nociceptive signal modulation remains unclear.

In the context of the general role of pain as a danger signal, it may be beneficial for TRN to be inhibited for acute nociception, for unimpeded nociceptive signal transmission. Indeed, inhibition of TRN neuronal activity to acute nociceptive stimulus has been demonstrated in a previous study done under anesthesia (Yen and Shaw, 2003). For sustained nociception, however, it may be more advantageous to attenuate the level of nociception because sustained nociception serves a different purpose, which is to protect the affected tissue. Adjusting the level of nociception, just enough to protect the affected tissue, may be more beneficial for sustained nociception because discomfort caused by prolonged pain can rather be debilitating. This may be why the activities of TRN neurons, especially typical TRN neurons, were significantly potentiated as shown in this study. Differential responses of TRN to acute and sustained nociception may be the hallmark of properly functioning TRN in nociceptive signal processing.

In case of thalamic pain syndromes which cause neuropathic pain, proper functioning of TRN neurons may be disrupted. Alterations in burst firing properties of sensory thalamic neurons have been reported in clinical cases and in an animal model of neuropathic pain (Lenz et al., 1989, 1994; Hains et al., 2006). In an animal model of neuropathic pain, burst firing properties of sensory thalamic neurons were altered to have shorter burst length, smaller number of burst spikes per burst, and longer IntraBIs (Hains et al., 2006). Since inhibitory TRN input could influence burst firing properties of sensory thalamic neurons, dysfunction of TRN may have contributed to the debilitating symptoms of neuropathic pain. Especially typical TRN neurons, which appears to have the greatest influence in controlling the activity of sensory thalamic neurons in this study, may have been more affected than other neuronal types in neuropathic pain.

Overall, this study showed that different TRN neuronal subtypes respond differentially to formalin induced nociception. Typical TRN neurons had the greatest modulation to nociception while the other neuronal subtypes showed less modulation.

## Author Contributions

YH and JC designed the experiment, analyzed the data and wrote the manuscript. YH carried out the experiments.

## Funding

This research was funded by the Ministry of Science, ICT and Future Planning (MSIP) through the National Research Foundation of Korea (NRF) grants: Mid-career Researcher Program (NRF-2015R1A2A2A04005487) and Brain Science Research Program (NRF-2015M3C7A1028392). This research was also funded by the Korea Institute of Science and Technology Intramural Funds (2E26640 and 2E26663).

## Conflict of Interest Statement

The authors declare that the research was conducted in the absence of any commercial or financial relationships that could be construed as a potential conflict of interest.
