# Supplementary figures and images for: Differential Responses of Thalamic Reticular Neurons to Nociception in Freely Behaving Mice
Source: Front Behav Neurosci. 2016 Nov 21;10:223. doi: 10.3389/fnbeh.2016.00223 (PMC5116476; doi:10.3389/fnbeh.2016.00223)

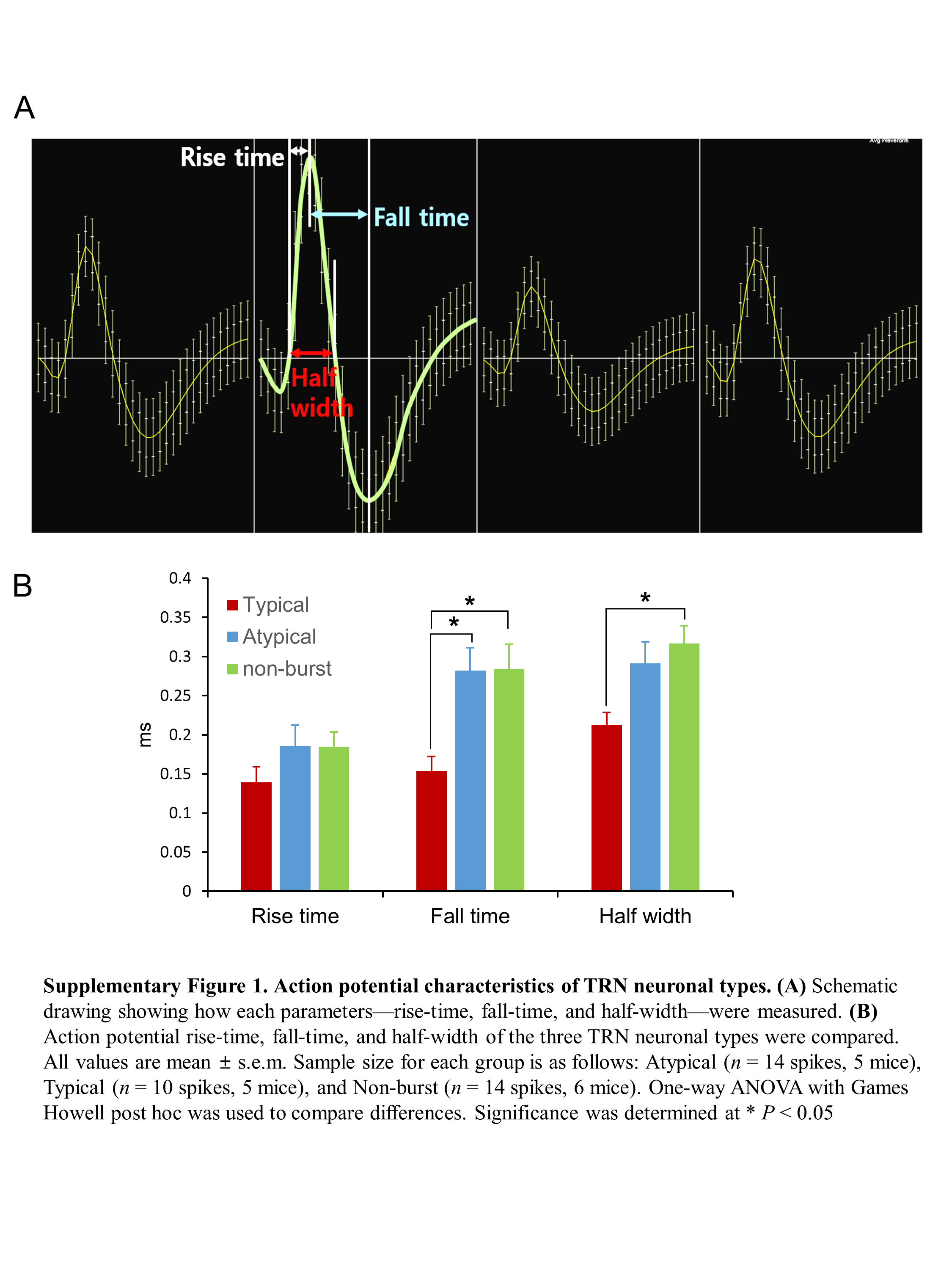

Supplement: Supplementary file 1 [file Image_1.tif]

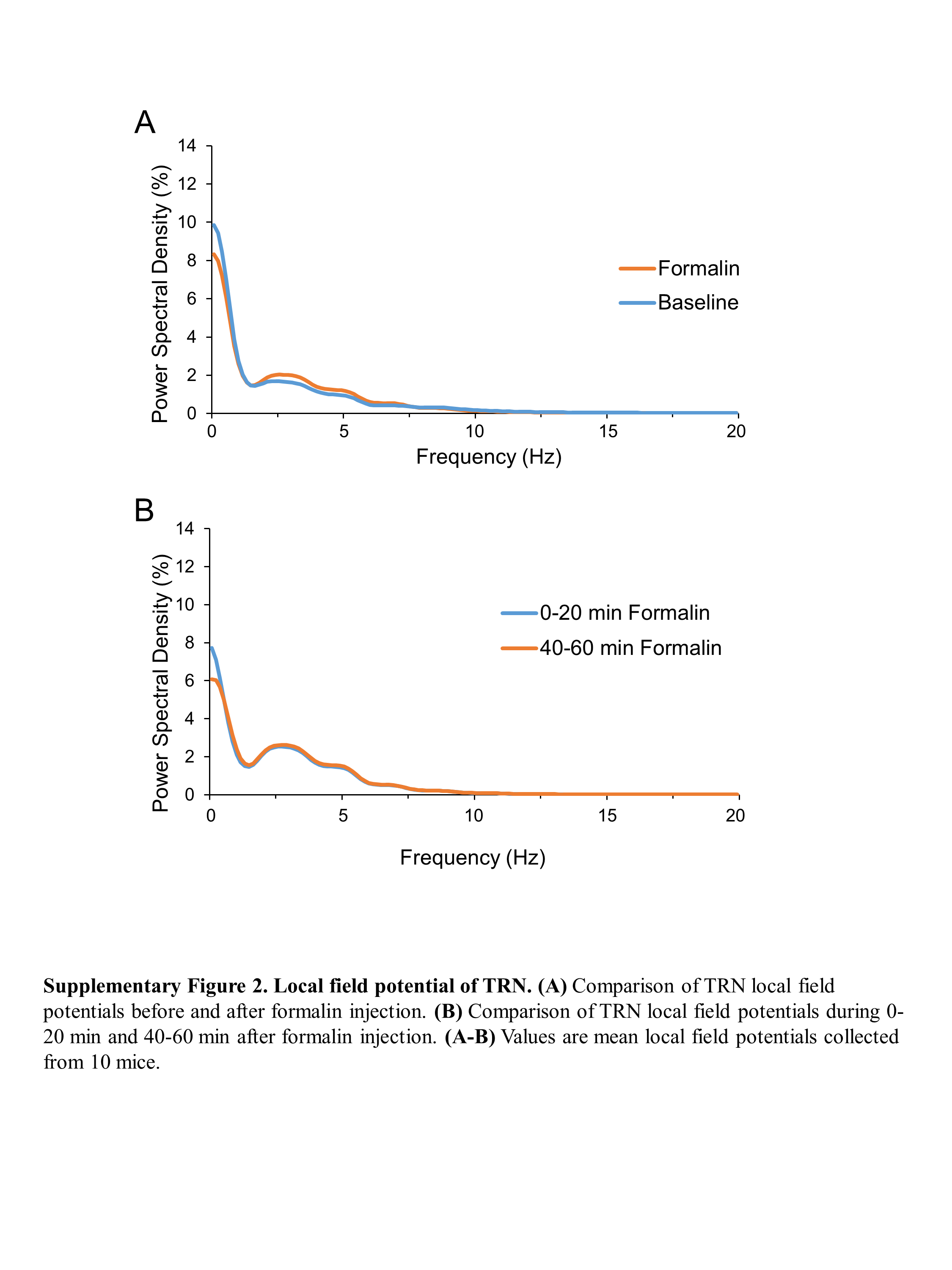

Supplement: Supplementary file 2 [file Image_2.tif]
